# Trajectory patterns of metabolic syndrome severity score and risk of type 2 diabetes

**DOI:** 10.1186/s12967-023-04639-w

**Published:** 2023-10-25

**Authors:** Atieh Amouzegar, Mohammadjavad Honarvar, Safdar Masoumi, Davood Khalili, Fereidoun Azizi, Ladan Mehran

**Affiliations:** 1 Endocrine Research Center, Research Institute for Endocrine Sciences, Shahid Beheshti University of Medical Sciences, No. 23, Parvaneh Street, Velenjak, Tehran, P.O. Box: 19395-4763 IR Iran; 2 Prevention of Metabolic Disorders Research Center, Research Institute for Endocrine Sciences, Shahid Beheshti University of Medical Sciences, Tehran, IR Iran; 3 Department of Biostatistics and Epidemiology, Research Institute for Endocrine Sciences, Shahid Beheshti University of Medical Sciences, Tehran, IR Iran

**Keywords:** Diabetes, Metabolic syndrome, Risk, Trajectory, Growth mixture model, Cardio metabolic risk factors

## Abstract

**Background:**

The available evidence indicates that the severity of metabolic syndrome tends to worsen progressively over time. We assessed the trajectory of age and sex-specific continuous MetS severity score (cMetS-S) and its association with the development of diabetes during an 18-year follow-up.

**Methods:**

In a prospective population-based Tehran Lipid and Glucose Study, 3931 eligible participants free of diabetes, aged 20–60 years, were followed at three-year intervals. We examined the trajectories of cMetS-S over nine years using latent growth mixture modeling (LGMM) and subsequent risks of incident diabetes eight years later. The prospective association of identified trajectories with diabetes was examined using the Cox proportional hazard model adjusting for age, sex, education, and family history of diabetes, physical activity, obesity (BMI ≥ 30 kg/m^2^), antihypertensive and lipid-lowering medication, and baseline fasting plasma glucose in a stepwise manner.

**Results:**

Among 3931 participants, three cMetS-S trajectory groups of low (24.1%), medium (46.8%), and high (29.1%) were identified during the exposure period. Participants in the medium and high cMetS-S trajectory classes had HRs of 2.44 (95% CI: 1.56–3.81) and 6.81 (95% CI: 4.07–10.01) for future diabetes in fully adjusted models, respectively. Normoglycemic individuals within the high cMetS-S class had an over seven-fold increased risk of diabetes (HR: 7.12; 95% CI: 6.05–12.52).

**Conclusion:**

Although most adults exhibit an unhealthy metabolic score, its severity usually remains stable throughout adulthood over ten years of follow-up. The severity score of metabolic syndrome has the potential to be utilized as a comprehensive and easily measurable indicator of cardiometabolic dysfunction. It can be employed in clinical settings to detect and track individuals at a heightened risk of developing T2DM, even if their glucose levels are normal**.**

**Supplementary Information:**

The online version contains supplementary material available at 10.1186/s12967-023-04639-w.

## Introduction

Type 2 diabetes mellitus (T2DM) is one of the major public health issues affecting more than 450 million adults worldwide[1]. The International Diabetes Federation's most recent report predicts a significant rise in the prevalence of diabetes, particularly in the Middle East and North Africa region, expected to reach 19.3% by 2045, despite ongoing measures to prevent and manage the disease [2]. The significant morbidity and mortality linked with T2DM underscores the emerging necessity for advancing research into the risks that predispose individuals to develop this condition. Metabolic syndrome (MetS), conceptualized based on a cluster of five cardiometabolic risk factors, has been strongly linked to five-fold and two-fold elevated risk of future T2DM and cardiovascular disease (CVD), respectively [3, 4]. Insulin resistance, neurohormonal activation, and chronic inflammation are contributory mechanisms of MetS and its transition to T2DM and CVD [5]. Accordingly, MetS can be regarded as a potential clinical tool that may facilitate the identification and ongoing surveillance of individuals at risk of T2DM and CVD.

The clinical applicability of MetS is constrained by its binary framework, which results in a loss of information regarding the severity of MetS factors and a failure to consider the spectrum of metabolic abnormality. The status of metabolic syndrome and its individual components may exhibit significant alterations over time, as elucidated by various cohort and interventional studies [6–9]; thus, tracking the evolving severity of MetS is likely to yield substantial utility in monitoring cardiometabolic derangement. Few research studies have elucidated the association between dynamic changes in MetS and the risk of diabetes compared to individuals only when their MetS status changes (developing or recovering from MetS), leading to low precision in predicting health outcomes [6, 10]. However, these investigations have primarily evaluated individuals solely upon the occurrence of alterations in their MetS status, either through its development or recovery, resulting in low precision in the prediction of health outcomes. It can be challenging to monitor changes in Mets' trends and their effects on health outcomes over time because some people may experience consistent MetS status, while others may switch between having or not having MetS.

To overcome these limitations, we developed age- and sex-specific continuous MetS severity score (cMetS-S) equations using confirmatory factor analysis (CFA) to reflect the severity of MetS, considering the weighted contribution of the MetS components to MetS [11]. In clinical practice, the cMetS-S can be used as an indicator of metabolic health in the primary care setting. Using the age- and sex-specific cMetS-S equations, each individual will have a MetS severity score, with higher values indicating higher MetS severity. In contrast to the dichotomous traditional MetS criteria (presence vs. absence), this age- and sex-specific cMetS-S score considers the degree of abnormality of the MetS components and tracks the changes in the severity of MetS. We aimed to classify the population according to time-serial cMetS-S trajectories and explore the association between the longitudinal trajectories of cMetS-S and future T2DM using latent growth mixture modeling during 18 years of follow-up in a prospective population-based cohort Tehran Lipid and Glucose Study (TLGS).

## Method

### Study population

This study was conducted within the Tehran Lipid and Glucose Study framework, a longitudinal population-based cohort conducted to investigate non-communicable diseases in Iran. Previous papers have described The TLGS design in detail [12]. TLGS was initiated by enrolling participants in the year 1999 and subsequently undertaking assessments of these participants at intervals of roughly three years over a span of eighteen years, comprising six follow-up periods.

**Fig. 1:**
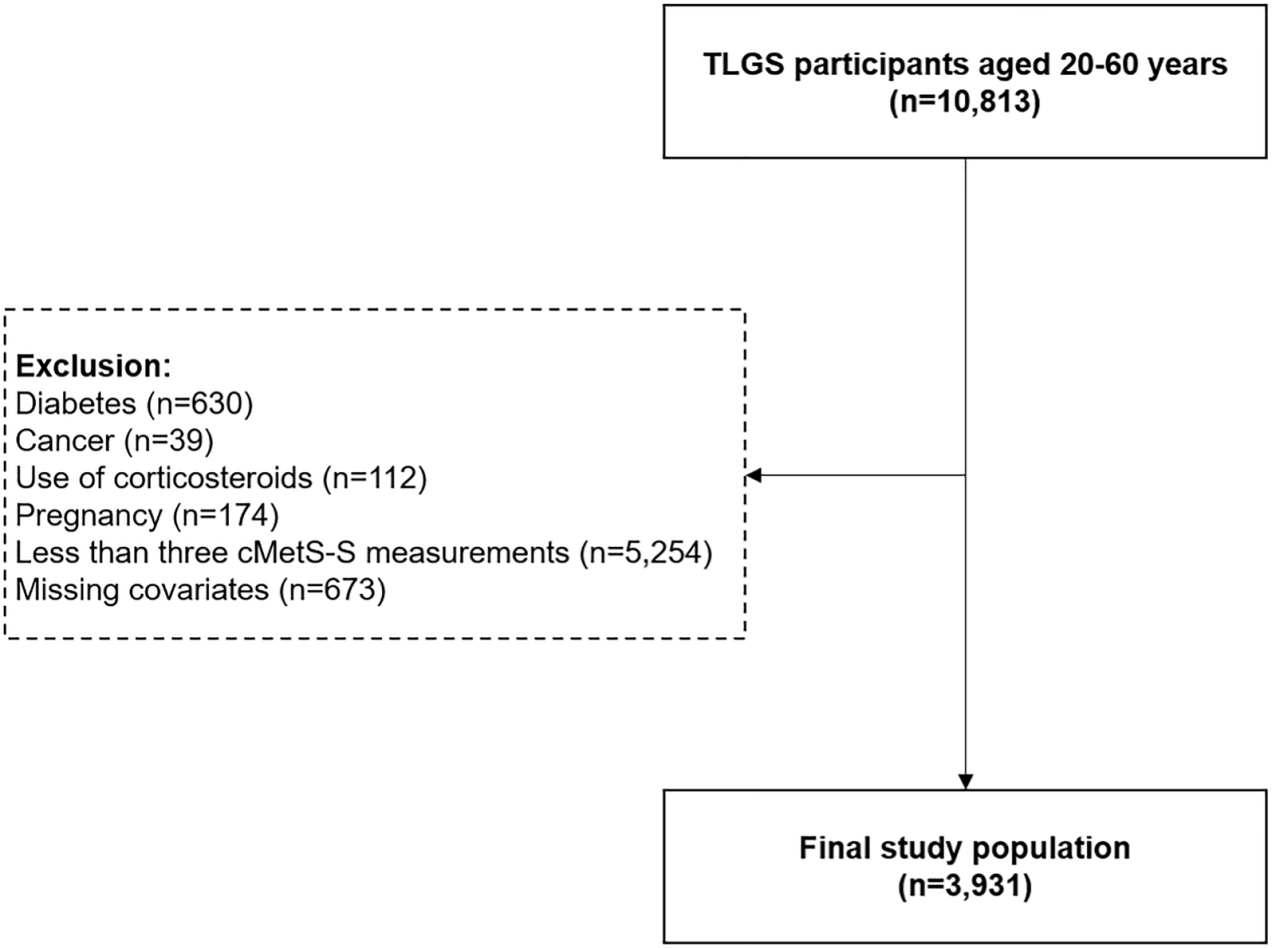
Flowchart illustrating participant selection. *TLGS* Tehran Lipid and Glucose Study, *cMetS-S* continuous metabolic syndrome severity score

**Fig. 2:**
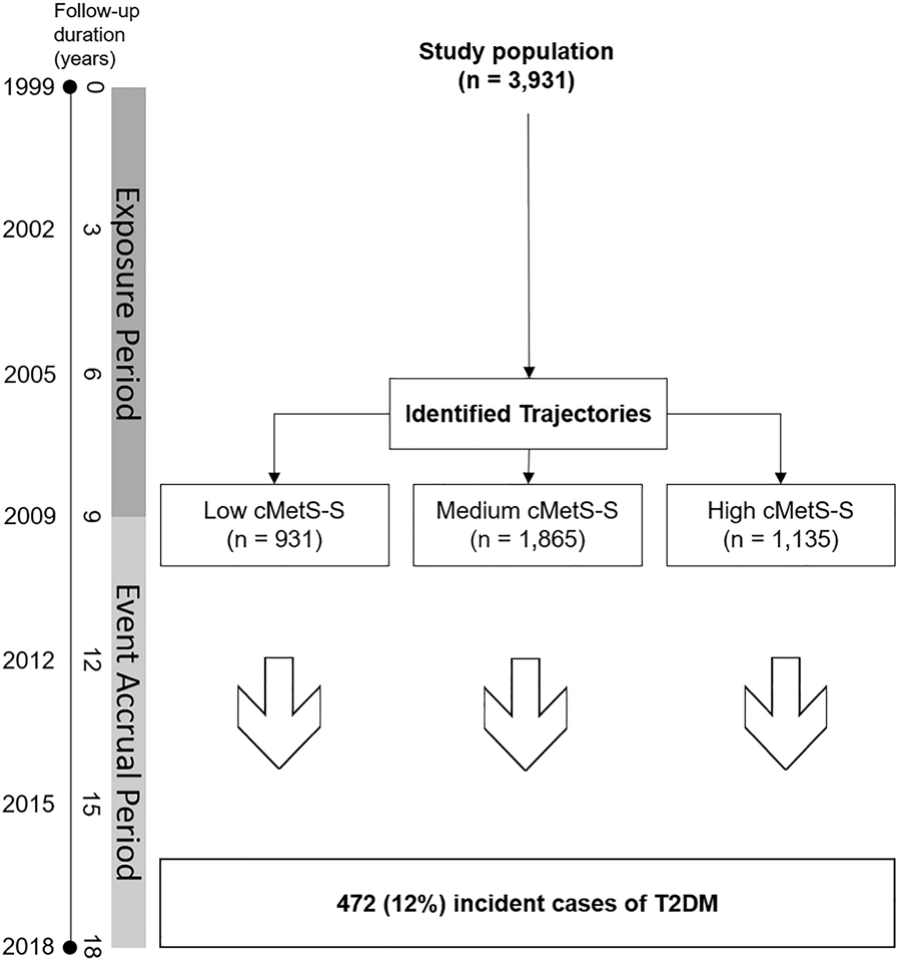
Analysis flow. The study population was classified into three trajectories of low, medium, and high cMetS-S in the exposure period and followed for the incidence of T2DM in the event accrual period. *cMetS-S* continuous metabolic syndrome severity score, *T2DM* type 2 diabetes mellitus

In the current study, the follow-up period was divided into two intervals: a 9-year exposure period for identifying cMetS-S trajectories (from the baseline to the end of the third follow-up visit) and a 9-year event accrual period for measuring diabetes incidence (from the third follow-up visit to the end of the study). A total of 10,813 participants aged 20–60 years were included in the study. Individuals with diabetes (n = 630), cancer (n = 39), use of corticosteroids (n = 112), pregnancy (n = 174), less than three cMetS-S measures in the exposure period (n = 5254), and missing data on covariates (n = 673) were excluded (Figs. 1 and 2). The occurrence of diabetes was assessed from the beginning of the event accrual period in three-year intervals. Diabetes was defined as one of the following: 1) fasting plasma glucose (FPG) level ≥ 126 mg/dl 2) 2-h post-challenge plasma glucose (2 h-PCP) ≥ 200 mg/dl) 3) treatment with diabetes medication.

### Data collection

Information on participants, including age, smoking status, education, physical activity, medical history, and medication, was collected through interview-administered questionnaires. Education level was classified into primary (6 years), high school (6–12 years), and higher (> 12 years) education. Physical activity was calculated based on the metabolic equivalent of the task scale (METS) using data derived from the physical activity questionnaires. Participants with a METS score of less than 600 min per week were considered to have low physical activity. The trained physicians obtained anthropometric measurements of waist circumference, weight, and height using standard protocols, and body mass index was calculated accordingly. The blood samples of the participants were drawn after a 12- to 14-h overnight fasting and analyzed for levels of FPG, triglycerides (TG), total cholesterol (TC), high-density lipoprotein cholesterol (HDL-C), and creatinine (Cr) on the same day using standard laboratory assays and techniques. Questionnaires and clinical and laboratory measurement protocols used in TLGS were previously described in detail [12]. Prediabetes was defined by one of the following: FPG value ranged from 100 to 126 mg/dl or 2h-PCPG in the range of 140–199 mg/dl, without using glucose‐lowering drugs. Individuals with FPG value < 100 mg/dL and 2h‐PCPG < 140 mg/dL were considered normal according to the American Diabetes Association definition. Hypertension was defined as SBP ≥ 140 mmHg, DBP ≥ 90 mmHg, or being on medication for hypertension. Dyslipidemia was considered when TG ≥ 150 mg/dl, TC ≥ 200 mg/dl, HDL < 40 mg/dl in men and < 50 mg/dl in women, or use of lipid-lowering drugs. The eGFR was obtained using the Chronic Kidney Disease Epidemiology Collaboration creatinine equation (CKD-EPI 2021).

### CMetS-S definition

The cMetS-S score was previously developed according to sex and age categories of 20–39 years and 40–60 years using CFA, considering the weighted contribution of MetS components in each sex and age category (Additional file 1: Table S1) [11]. cMetS-S was standardized at mean = 0 and SD = 1 for easy interpretation. The resulting cMetS-S is a z-score, with higher scores indicating higher MetS severity.

### Statistical analysis

We analyzed cMetS-S trajectories in the exposure period using latent growth mixture modeling (LGMM) within the "lcmm" package in R software. Random intercept models were utilized, and the participants' follow-up visits were included as an independent variable to better understand the course of cMetS-S over time. Visit-cubic natural linear models were used to assess the potential nonlinearity of risk factors over time, with a maximum of four nodes in the fixed-effect part. The number of route groups was limited to a maximum of five groups, and model fitness was evaluated using Bayesian Information Criterion, posterior probability (> 0.7), average likelihood of group membership, and root mean error of approximation (RMEA). To ensure clinical meaningfulness, each class was required to include at least 5% of the participants, and models with fewer than 5% of the participants were discarded. Trajectories were identified in total and also separately in normoglycemic and prediabetic participants.

The association of cMetS-S trajectory classes with incident T2DM in the event accrual period was determined using trajectory class membership as an independent variable. This association was calculated as hazard ratios (HRs) and 95% confidence intervals, with the low cMetS-S trajectory as the reference group using multivariate Cox proportional hazard regression models. Graphical checks based on log–log plots of survival and tests based on Schoenfeld residuals were used to examine the proportional hazards assumption. We stratified the Cox models on covariates that did not satisfy the proportional hazards assumption. The method of entering covariate or confounding variables into the model was "Enter." So, all the variables that were considered confounding in terms of the literature review and experts' views were included in four models. Model 1 was adjusted for age and sex. Model 2 was adjusted for age, sex, education, and family history of diabetes. Model 3 was additionally adjusted for physical activity, obesity (BMI ≥ 30 kg/m^2^), antihypertensive and lipid-lowering medication. Model 4 was adjusted for the covariates in model 3 and baseline FPG. For each participant, we calculated the person-time of follow-up starting from the completion of the third cMetS-S measurement and ending at the earliest occurrence of the following events: diabetes diagnosis, death, loss to follow-up, or the end of the follow-up period. The statistical analyses were performed with STATA 14 (StataCorp, college station, TX, USA) and R-3.0.3 (R Foundation for Statistical Computing, Vienna, Austria). A two-sided P-value < 0.05 was considered statistically significant.

## Results

**Fig. 3:**
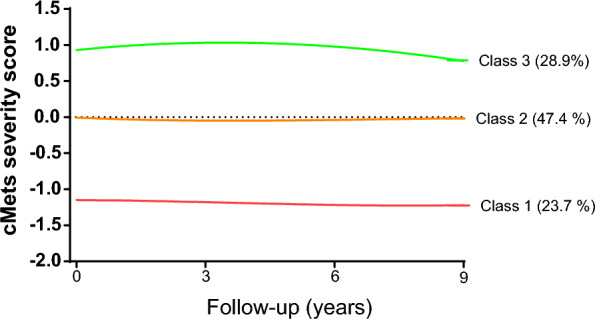
Identified trajectory patterns of cMetS-S severity score during the exposure period. The population was classified into three trajectory classes using latent growth mixture modeling, including trajectories of low (class 1), medium (class 2), and high (class 3) cMetS-S. *cMetS-S* continuous metabolic syndrome severity score

The study population included 3931 participants (41.9% men) with a mean age of 38.1 ± 10.58 years at baseline (Table 1). We categorized the population into three groups based on cMetS-S trajectory classes. The identified cMetS-S trajectories during the exposure period were low (class 1), medium (class 2), and high (class 3) (Fig. 3). The proportions of individuals in the low, medium, and high cMetS-S trajectories were 24.1%, 46.8%, and 29.1%, respectively.

**Table 1:** Baseline characteristics of the study population according to continuous metabolic syndrome severity score (cMetS-S) trajectory classes

Characteristics	Overall	cMetS-S* trajectory patterns			P-value
		Class 1	Class 2	Class 3	
Number of participants	3931	931	1865	1135	–
Age (years)	38.08 ± 10.58	32.61 ± 9.74	38.60 ± 10.25	41.71 ± 9.98	< 0.001
Male	1648 (41.92)	202 (21.70)	813 (43.59)	633 (55.77)	< 0.001
Body mass index (kg/m2)	26.70 ± 4.51	23.68 ± 3.73	26.69 ± 4.09	29.18 ± 4.23	< 0.001
Waist circumference (cm)	87.25 ± 11.56	77.56 ± 9.23	87.22 ± 9.77	95.24 ± 9.76	< 0.001
Education					< 0.001
Illiterate/primary school (< 6 yrs.)	2355 (59.91)	502 (53.92)	1129 (60.54)	724 (63.79)	
High school (6–12 years)	985 (25.06)	253 (27.18)	467 (25.04)	265 (23.35)	
Higher education (≥ 12 years)	591 (15.03)	176 (18.90)	269 (14.42)	146 (12.86)	
Smokers	490 (12.47)	64 (6.87)	240 (12.87)	186 (16.39)	< 0.001
Low physical activity	2637 (67.08)	612 (65.74)	1250 (67.02)	775 (68.28)	0.47
DM family history	363 (9.89)	85 (9.70)	178 (10.15)	100 (9.59)	0.52
Hypertension	291 (25.64)	291 (25.64)	291 (25.64)	291 (25.64)	< 0.001
Dyslipidemia	1679 (42.71)	20 (2.15)	665 (35.66)	994 (87.58)	< 0.001
SBP (mmHg)	114.43 ± 15.43	106.28 ± 11.79	114.01 ± 14.29	121.82 ± 16.31	< 0.001
DBP (mmHg)	76.38 ± 10.21	71.30 ± 8.75	76.14 ± 9.62	80.94 ± 10.20	< 0.001
FBS (mg/dL)	88.46 ± 8.73	84.98 ± 7.72	88.59 ± 8.44	91.12 ± 9.00	< 0.001
Triglyceride (mg/dL)	157.51 ± 97.40	80.89 ± 27.45	138.35 ± 51.05	251.85 ± 116.81	< 0.001
HDL-C (mg/dL)	42.06 ± 10.78	50.07 ± 11.02	41.99 ± 9.18	35.60 ± 8.38	< 0.001
Antihypertensive drug use	116 (2.95)	6 (0.64)	55 (2.95)	55 (4.85)	< 0.001
Lipid-lowering drug use	52 (1.32)	2 (0.21)	19 (1.02)	31 (2.73)	< 0.001
MetS (JIS)	1123 (28.57)	7 (0.75)	338 (18.12)	778 (68.55)	< 0.001
MetS (IDF)	1000 (25.44)	6 (0.64)	281 (15.07)	713 (62.82)	< 0.001
cMetS-S	0.0 ± 1.0	-1.20 ± 0.56	− 0.05 ± 0.55	1.07 ± 0.59	< 0.001

*The population was classified into three trajectory classes using latent growth mixture modeling, including trajectories of low (class 1), medium (class 2), and high (class 3) cMetS-S

The categorical and continuous variables were reported as count (percentage) and mean ± SD, respectively

*cMetS-S* continuous metabolic syndrome severity score, *DM* diabetes, *SBP* systolic blood pressure, *DBP* diastolic blood pressure, *FBS* fasting blood sugar, *HDL-C* high-density lipoprotein cholesterol, *MetS* metabolic syndrome, *JIS* joint interim statement, *IDF* International Diabetes Federation

The mean values of age, BMI, and all MetS components (except HDL) were significantly greater in the high cMetS-S trajectory class. The prevalence of men, smokers, and individuals with hypertension and dyslipidemia was also higher in the high cMetS-S trajectory class (P value < 0.001) compared to the low cMetS-S trajectory class. There was no difference in the prevalence of low physical activity or diabetes family history across the cMetS-S trajectory patterns. The baseline characteristics of the population are also presented according to subgroups of prediabetic and normoglycemic subjects in Additional file 1: Table S2.

During 18 years of follow-up, we recorded 472 (12%) incident cases of diabetes. The incidence rate of diabetes was 2.8 (95% CI 1.8–4.2) in low, 10.1 (95% CI 8.7–11.8) in medium, and 30.4 (95% CI: 27.0–34.2) in high cMetS-S trajectory classes, respectively (Table 2). Membership in medium and high cMetS-S trajectory patterns significantly increased the risk of diabetes with HRs of 2.44 (95% CI: 1.56–3.81) and 6.81 (95% CI: 4.07–10.01) in fully adjusted models, respectively.

**Table 2:** Cox proportional hazard ratios (HRs) of the identified cMetS-S trajectory patterns for incidence T2DM in total and according to sex

	Events	IR (95% CI)^*^	HR (95% CI)				Test PH p-value^b^
			Model 1	Model 2	Model 3	Model 4	
Men (n = 1648)							
Class 1 (n = 202)	4	2.3 (0.9–6.1)	1.0 (Reference)	1.0 (Reference)	1.0 (Reference)	1.0 (Reference)	–
Class 2 (n = 813)	68	9.5 (7.5–12.0)	3.70 (1.35–10.18)	3.70(1.35–10.18)	3.36(1.22–9.25)	2.83(1.03–7.81)	0.6
Class 3 (n = 633)	129	24.6 (20.7–29.3)	10.22 (3.77–27.68)	10.20(3.77–27.63)	8.90(3.27–24.25)	6.80(2.48–18.58)	0.98
Women (n = 2283)							
Class 1 (n = 729)	19	2.9 (1.9–4.6)	1.0 (Reference)	1.0 (Reference)	1.0 (Reference)	1.0 (Reference)	–
Class 2 (n = 1052)	100	10.6 (8.7–12.9)	3.31 (2.00–5.45)	3.33(2.02–5.49)	3.10(1.86–5.11)	2.52(1.51–4.18)	0.07
Class 3 (n = 502)	152	37.9 (32.3–44.4)	11.95 (7.20–19.85)	12.11(7.29–20.12)	10.63(6.33–17.86)	7.65(4.54–12.89)	0.13
Total (n = 3931)							
Class 1 (n = 931)	23	2.8 (1.8–4.2)	1.0 (Reference)	1.0 (Reference)	1.0 (Reference)	1.0 (Reference)	–
Class 2 (n = 1865)	168	10.1 (8.7–11.8)	3.13 (2.01–4.87)	3.10(1.99–4.84)	2.87(1.84–4.48)	2.44(1.56–3.81)	0.08
Class 3 (n = 1135)	281	30.4 (27.0–34.2)	9.58 (6.17–14.87)	9.47(6.10–14.71)	8.37(5.35–13.09)	6.38(4.07–10.01)	0.25

Model 1: Adjusted for age and sex

Model 2: Adjusted for age, sex, education, DM family history

Model 3: Adjusted for age, sex, education, DM family history, physical activity, obesity, antihypertensive drug use, lipid-lowering drug use

Model 4: Adjusted for age, sex, education, DM family history, physical activity, obesity, antihypertensive drug use, lipid-lowering drug use. and fasting plasma glucose

The population was classified into three trajectory classes using latent class analysis, including trajectories of low (class 1), medium (class 2), and high (class 3) cMetS-S

*cMetS-S* continuous metabolic syndrome severity score;

^a^*IR* Incidence rate per 1,000 person-years

^b^The proportional hazards assumption was tested based on Schoenfeld residuals.There is no evidence that proportional-hazards assumption has been violated. In the fully adjusted model (model 4), the p-value of the global test proportional hazards assumption is equal to 0.37

**Fig. 4:**
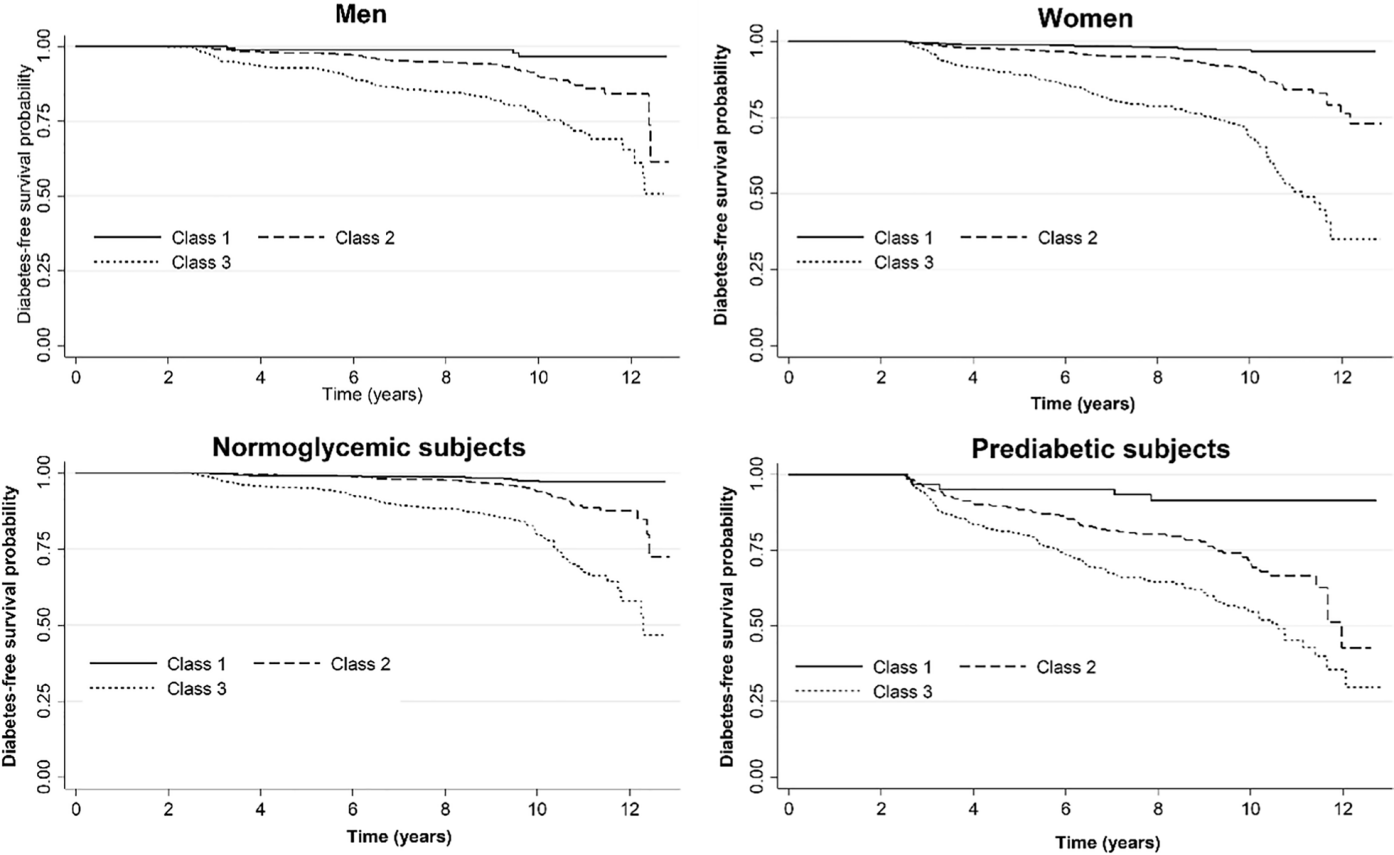
Kaplan–Meier curve of diabetes-free probability by cMetS-S trajectories in different subgroups. The population was classified into three trajectory classes using latent class analysis, including trajectories of low (class 1), medium (class 2), and high (class 3) cMetS-S. *cMetS-S* continuous metabolic syndrome severity score

In subgroup analyses, the medium and high cMetS-S trajectory class had an incrementally higher risk of incident diabetes than the low cMetS-S trajectory class (Tables 2 and 3). The high cMetS-S trajectory pattern had an HR of 6.80 (95% CI: 2.48–18.58) in men and an HR of 7.56 (95% CI: 4.54–12.89) in women (Table 2). Another subgroup analysis was conducted in prediabetic and normal subjects (Table 3). The high cMetS-S pattern was associated with future diabetes in prediabetics (HR:3.78; 95% CI:2.15–6.65) and normoglycemic subjects (HR:7.12; 95% CI:6.05–12.52). The Kaplan–Meier curve demonstrated a significant difference in diabetes-free probability between cMetS-S–S trajectory patterns, with medium and high trajectories having poorer diabetes-free probability in all subgroups (P log rank < 0.001) (Fig. 4).

**Table 3:** Cox proportional hazard ratios (HRs) of the identified cMetS-S trajectory patterns for incidence type 2 diabetes mellitus in normoglycemic and prediabetic individuals

	Events	IR (95% CI)^a^	HR (95% CI)				Test PH p-value^b^
			Model 1	Model 2	Model 3	Model 4	
Normoglycemic subjects at baseline (n = 3260)							
Class 1 (n = 867)	15	2.1 (1.2–3.4)	1.0 (Reference)	1.0 (Reference)	1.0 (Reference)	1.0 (Reference)	–
Class 2 (n = 1558)	83	6.1 (4.9–7.6)	2.67 (1.53–4.68)	2.64 (1.51–4.62)	2.39 (1.36–4.21)	2.20 (1.25–3.88)	0.41
Class 3 (n = 835)	159	20.1 (17.2–23.4)	9.46 5.44–16.43)	9.24 (5.31–16.07)	7.93(4.51–13.94)	7.12 (6.05–12.52)	0.78
Prediabetic subjects at baseline (n = 671)							
Class 1 (n = 64)	16	15.6 (9.6–25.5)	1.0 (Reference)	1.0 (Reference)	1.0 (Reference)	1.0 (Reference)	–
Class 2 (n = 307)	94	37.8 (30.9–46.3)	2.54 (1.46–4.41)	2.51 (1.45–4.37)	2.60 (1.50–4.52)	2.53 (1.46–4.66)	0.09
Class 3 (n = 300)	105	60.6 (50.1–73.4)	4.04 (2.32–7.03)	3.74 (2.13–6.56)	3.99 (2.27–7.00)	3.78 (2.15–6.65)	0.14

Model 1: Adjusted for age and sex

Model 2: Adjusted for age, sex, education, DM family history

Model 3: Adjusted for age, sex, education, DM family history, physical activity, obesity, antihypertensive drug use, lipid-lowering drug use

Model 4: Adjusted for age, sex, education, DM family history, physical activity, obesity, antihypertensive drug use, lipid-lowering drug use. and fasting plasma glucose

The population was classified into three trajectory classes using latent class analysis, including trajectories of low (class 1), medium (class 2), and high (class 3) cMetS-S

CMetS-S, continuous metabolic syndrome severity score; PH, proportional-hazards assumption

^***a***^***IR*** Incidence rate per 1,000 person-years

^b^The proportional hazards assumption was tested based on Schoenfeld residuals. There is no evidence that proportional-hazards assumption has been violated (p > 0.5). In the fully adjusted model (model 4), the p-value of the proportional hazards assumption of the global test in normoglycemic and prediabetic subjects at baseline is equal to 0.18 and 0.56, respectively

## Discussion

This groundbreaking research presents a unique depiction of how the severity of metabolic syndrome changes over time and how it relates to the development of type 2 diabetes in a representative sample of Iranian adults involved in a long-term population-based cohort study spanning 18 years. Using the GMM trajectory modeling technique on a longitudinal dataset of MetS severity scores across three examination cycles helps us to identify discrete hidden growth patterns of MetS severity scores by illustrating particular sets of people who exhibit distinctive MetS severity score trends throughout the observed periods. The cMetS-S trajectory during adulthood displayed three distinct patterns; notably, the severity of MetS remained relatively stable within each pattern over the 10-year observation period. The demographic segment comprising individuals with middle and high trajectory patterns constituted more than three-quarters of the overall population. The risk of diabetes increased by more than twice in individuals following the middle cMetS-S trajectory pattern and over six times in those following the high cMetS-S trajectory pattern, even after excluding those who were prediabetic, as per the fully adjusted model.

Many epidemiological studies have indicated the association between the different sets of the traditional MetS criteria and new-onset T2DM; however, most evidence casts substantial doubt on its clinical predictive value beyond its individual components [3, 13, 14]. There still has been much controversy concerning the certainty of the definition and its value in identifying those at high risk of diabetes [3, 15]. MetS as a whole entity encounters many drawbacks in clinical practice, e.g., different definitions impede the compelling necessity to precisely detect individuals at risk for health outcomes [15]. While MetS covers the wide spectrum of metabolic abnormality, its binary criteria overlooks the severity of each component and the exerted load on β cell function and limits tracking the changes made over time. Although evidence indicates that obesity and impaired glucose regulation are the most potent predictors of diabetes, MetS assigns similar risk estimates to each component [16]. Also, a number of MetS-free individuals with probable risk for diabetes, such as prediabetics, might be missed. Creating a continuous scale from MetS components reflects the continuum of risk in MetS, which overcomes the above-mentioned limitations and improves the predictive ability of MetS by eluding loss of power from a dichotomous use of data.

Despite recent growing interest in MetS severity score, the studies in the Middle East are limited. A few reports suggest that the MetS severity z-score yields a further prediction of the development of diabetes over individual MetS components and is a stronger predictor than MetS [8, 17, 18]. Despite the ability of the score to track long-term metabolic changes and their health effects, no studies have evaluated the effect of the dynamic course of MetS severity score on diabetes and other health outcomes worldwide. Data from pulled cohorts of the Atherosclerosis Risk in Communities Study (ARIC) and the Jackson Heart Study (JHS) on 13,094 participants showed that MetS severity score at baseline was associated with future diabetes (HR = 17.4), which attenuated to the HR of 3.69 after adjusting for the individual MetS components [18]. The study by Gurka et al. lacked the assessment of the correlation between the trajectory of the MetS severity score and the onset of diabetes; nevertheless, a subsample analysis adjusted for age alone indicated that a MetS score increase of at least 0.05 over a 4-year period led to a 2.6-fold higher risk [18, 19]. Another study by the same group showed that adding MetS severity score to the common T2DM risk scores improved the discrimination and predictive performance of common T2DM risk scores [17] with much clearer clinical accuracy for T2DM than for CVD [17]. In a study by DeBoer et al., baseline MetS severity Z-score values predicted future diabetes, and the change in this score from adolescence to adulthood conveyed further disease risk and showed modest within-person durability of MetS severity over 26 years [20].

The high cMetS-S pattern was associated with a sevenfold increase in future diabetes in adults with normal blood glucose levels compared with a 3.7 increased risk in prediabetic individuals. This finding is of utmost importance as it highlights that adults with high MetS severity scores and normal blood glucose levels who are at high risk for future diabetes might be overlooked. The importance of screening for T2DM goes beyond just detecting people who are at prediabetic state; however, the ADA guideline recommends beginning screening in asymptomatic individuals without any risk factors at the age of 35 years and at three-year intervals [21]. In line with this finding, the recently released report suggests that the term prediabetes is problematic and that it is better to be replaced with more comprehensive and multifactorial risk models [22]. These models should incorporate various measures of glycemia, as well as sociodemographic and clinical information, to allow for the development of optimal intervention strategies tailored to each individual.

The time-serial clustering analysis indicated no substantial change in the categories of MetS severity as overall individuals with high MetS severity remained high and those with low MetS severity remained low in three assessments over ten years follow-up. This finding shows that poor metabolic health condition in adults remains stable over time, indicating that changes in health behavior tend to be more burdensome in adults. On the other hand, the stable longitudinal trend in cMetS-S might indicate a lack of timely preventive measures leading to better health status, especially in low- and middle-income countries. Although the evidence indicated positive effects of lifestyle intervention to prevent MetS, the effects disappeared after a few years and were not long-lasting [23–25]. In line with the current study, the Ewha Birth and Growth Study showed three distinct patterns in cMetS-S trajectory during childhood (3–12 years of age), and the cMetS-S score was consistently maintained throughout the observation period in all patterns (21). Koohi et al. detected three CVD risk score trajectories of high-increasing, medium-increasing, and low-stable during ten years; however, after keeping the age constant at baseline, the status of CVD risk score trajectories was similar but not increasing at the level of risk during the time indicating the impact of age on CVD risk score [26]. Bress and colleagues have also demonstrated the influence of aging on risk assessment scoring [27]. Based on the anticipated degradation of metabolic parameters during the aging process, the unchanging severity score trajectories of MetS appear illogical. However, this should be attributed to the age and gender-specific features of controlled (cMetS-S integrated into the model, wherein an individual's age is taken into account.

**Fig. 5:**
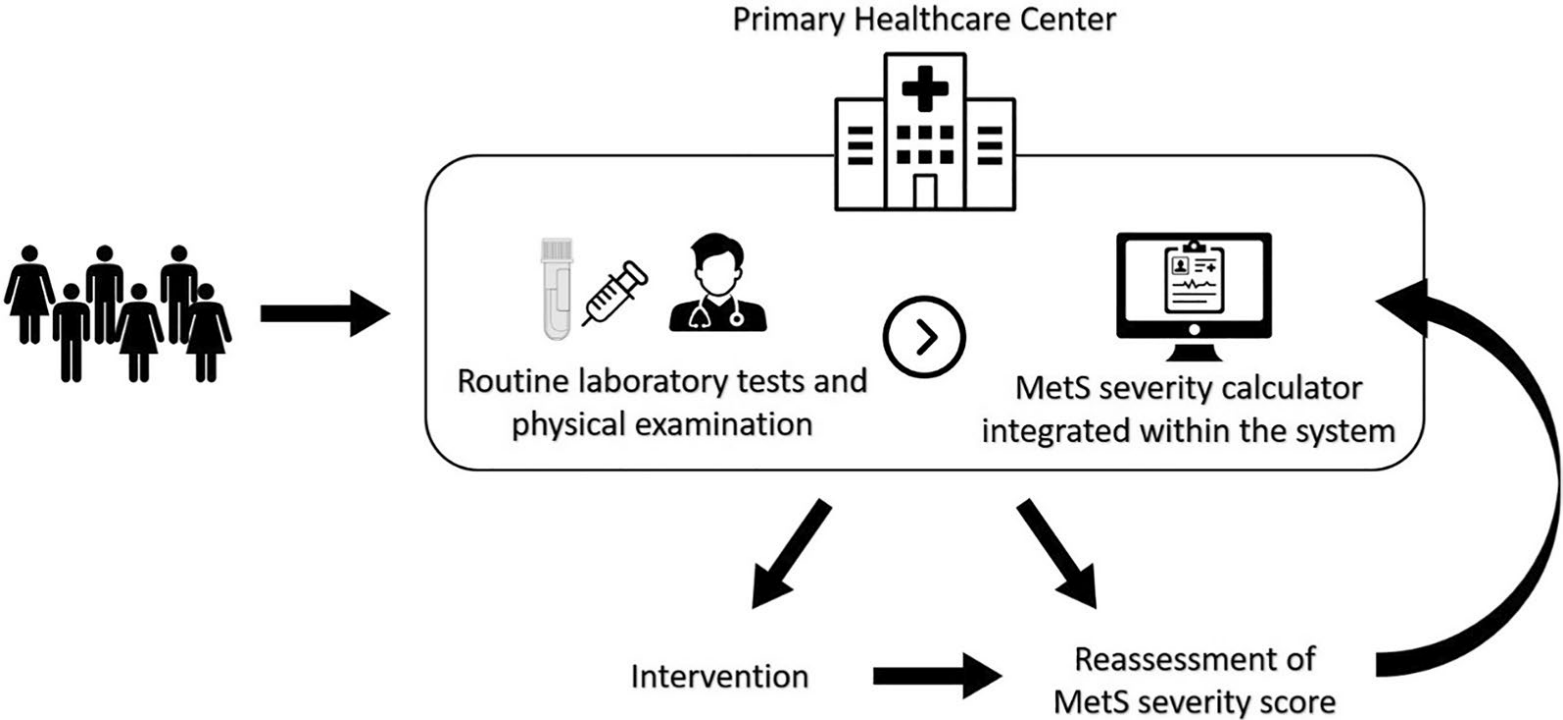
Assessment and management of metabolic syndrome using MetS severity score in the primary healthcare center. Laboratory measurement data, including triglyceride, high-density lipoprotein cholesterol, fasting plasma glucose levels, and data obtained from physical examination, including systolic blood pressure and waist circumference, are collected and entered into the electronic health record system. The integrated continuous metabolic syndrome severity score (cMetS-S) calculator utilizes the collected data to calculate the severity of metabolic syndrome based on the individual's sex and age category (20–39 and 40–60 years) using the cMetS-S equations. Recommendations and interventions are provided to individuals if necessary, and they are scheduled for reassessment for their next visit. *MetS* Metabolic syndrome

The MetS severity score equations which are formulated using CFA, vary across ethnicities due to the differences in the prevalence of MetS components contributing to MetS [11]. The only study on the association between MetS severity score and diabetes in the Iranian population suggested that each unit increase in baseline MetS severity score is associated with nearly two-fold increased risk of diabetes in the total population and women [28]. However, the study did not find a significant association in men. The reason may be that the MetS severity score was calculated based on the equations derived from the American white population instead of the Iranian population. The current study used age- and sex-specific MetS severity equations derived from the Iranian population. The absence of health policies focused on MetS, especially in low- and middle-income countries such as Iran underscores a critical gap in addressing the growing health challenges associated with MetS. The current study on MetS severity score reveals that individuals with a high severity score for MetS tend to have a consistently high score over the long term, regardless of their glycemic status. This finding highlights that the MetS severity score can be utilized to establish policies that begin with primary care, allowing for the regular assessment of individuals' metabolic health, monitoring of changes, and implementing interventions to prevent diabetes (Fig. 5).

This study exhibits notable strengths in terms of research methodology, data interpretation, and analytical framework employed in the investigation. The current investigation is distinguished from previous research endeavors in that it is the sole global study to assess the correlation between the trajectory of Metabolic Syndrome severity score and the incidence of T2DM. Moreover, the present research pertains to a population-based prospective investigation conducted on a representative sample of the Iranian population, consisting of individuals over 20 years of age during two decades of follow-up. Third, sophisticated GMM trajectory analysis enables us to classify populations to unobserved (latent) subpopulations with similar behavior or trajectory patterns, estimating an average growth curve for each class while allowing for variations between individuals of the same class by introducing random effects in the model. Other technical aspects, such as missing data, correlated residuals, and treating residuals in regressions and random effects in mixed effects models as latent variables, are all captured through GMM. Lastly, the model was stepwise adjusted for important confounders. The study's findings may not apply universally to all ethnic groups due to limited generalizability, given the evidence that highlights variations in the severity score of metabolic syndrome across different races [19]. The trajectory approach utilized in this study to collect data over three examination cycles may have omitted a small proportion of individuals (i.e., less than 5%) who exhibited improving or worsening patterns due to lifestyle modifications, resulting in the incorrect classification of these individuals into an inappropriate trajectory pattern.

We conclude that metabolic health conditions of the adult population, as indicated by cMetS-S, exhibit relative stability over the course of ten years. People with unhealthy metabolic states for over ten years are at a higher risk, more than eightfold, of developing diabetes in the future despite having presently normal blood glucose. The suggested ADA guideline of a three-year interval for screening healthy adults may require reassessment and evaluation [29]. It may be beneficial for these people to have regular check-ups and consider taking preventive actions like modifying their lifestyle. Smartly integrating an automatic calculation of the cMetS-S into an electronic health record (EHR) system would facilitate the process for healthcare providers to identify and monitor individuals at high risk, implement necessary treatment, and monitor their recovery progress.

### Supplementary Information

**Additional file 1: Table S1.** Age- and sex-specific equations of continuous metabolic syndrome severity score (cMetS-S) derived from confirmatory factor analysis. **Table S2.** Baseline characteristics of the study population based on prediabetic and normoglycemic subgroups.

## Data Availability

Datasets generated during and/or analyzed during the current study are not publicly available due to institutional policies but are available from the corresponding author on reasonable request.

## Abbreviations

ADA: American Diabetes Association
ARIC: Atherosclerosis Risk in Communities Study
BMI: Body mass index
CFA: Confirmatory factor analysis
CKD-EPI: Chronic Kidney Disease Epidemiology Collaboration
CMetS-S: Continuous MetS severity score
Cr: Creatinine
CVD: Cardiovascular Disease
DBP: Diastolic blood pressure
EHR: Electronic health record
eGFR: Estimated glomerular filtration rate
FPG: Fasting plasma glucose
GMM: Growth mixture modeling
HDL: High-density lipoprotein cholesterol
JHS: Jackson Heart Study
LGMM: Latent growth mixture modeling
LGMM: Latent growth mixture modeling
METS: Metabolic equivalent of the task scale
MetS: Metabolic syndrome
RMEA: Root mean error of approximation
SBP: Systolic blood pressure
TC: Total cholesterol
TG: Triglycerides
TLGS: Tehran Lipid and Glucose Study
T2DM: Type 2 diabetes mellitus
