# Supplementary material for: Trajectory patterns of metabolic syndrome severity score and risk of type 2 diabetes
Source: J Transl Med. 2023 Oct 25;21:750. doi: 10.1186/s12967-023-04639-w (PMC10598905; doi:10.1186/s12967-023-04639-w)
Supplement: Supplementary file 1 — Additional file 1: Table S1. Age- and sex-specific equations of continuous metabolic syndrome severity score (cMetS-S) derived from confirmatory factor analysis. Table S2. Baseline characteristics of the study population based on prediabetic and normoglycemic subgroups. [file 12967_2023_4639_MOESM1_ESM.docx]

**Additional file 1: Table S1.** Age- and sex-specific equations of continuous metabolic syndrome severity score (cMetS-S) derived from confirmatory factor analysis

| **Sex** | **Age** | **cMetS-S Equations** |
| --- | --- | --- |
| Male | 20 – 39 yr. | -1.79 + 0.24 × ln (TG) + 0.0045 × WC + 0.0017 × FPG + 0.0016 × SBP - 0.0042× HDL-C |
|  | 40 – 60 yr. | -1.67 + 0.25 × ln (TG) + 0.0034 × WC + 0.0014 × FPG + 0.0007 × SBP - 0.0042× HDL-C |
| Female | 20 – 39 yr. | -2.43 + 0.28 × ln (TG) + 0.0066 × WC + 0.0040 × FPG + 0.0039 × SBP - 0.0052 × HDL-C |
|  | 40 – 60 yr. | -2.37 + 0.41 × ln (TG) + 0.0021 × WC + 0.0015 × FPG + 0.0010 × SBP - 0.0040 × HDL-C |

Abbreviations: cMetS-S, continuous metabolic syndrome severity score; TG, triglyceride; WC, waist circumference; FPG, fasting plasma glucose; SBP, systolic blood pressure; HDL-C, high-density lipoprotein cholesterol

**Additional file 1: Table S2.** Baseline characteristics of the study population based on prediabetic and normoglycemic subgroups

| **Characteristics** |  | **Overall** |  | **Normoglycemic** |  | **Prediabetic** |  | **P‑value** |
| --- | --- | --- | --- | --- | --- | --- | --- | --- |
| Number of participants |  | 3931 |  | 3260 |  | 671 |  | - |
| Age (years) |  | 38.08±10.58 |  | 37.02±10.49 |  | 43.22±9.50 |  | <0.001 |
| Male |  | 1648 (41.92) |  | 1363 (41.81) |  | 285 (42.47) |  | 0.75 |
| Body mass index (kg/m^2^) |  | 26.70±4.51 |  | 26.39±4.44 |  | 28.19±4.53 |  | <0.001 |
| Waist circumference (cm) |  | 87.25±11.56 |  | 86.32±11.51 |  | 91.80±10.70 |  | <0.001 |
| Education |  |  |  |  |  |  |  | 0.019 |
| Illiterate/primary school (<6 yrs.) |  | 2355 (59.91) |  | 1922 (58.96) |  | 433 (64.53) |  |  |
| High school (6-12 years) |  | 985 (25.06) |  | 830 (25.46) |  | 155 (23.10) |  |  |
| Higher education (≥12 years) |  | 591 (15.03) |  | 508 (15.58) |  | 83 (12.37) |  |  |
| Smokers |  | 490 (12.47) |  | 415 (12.73) |  | 75 (11.18) |  | 0.26 |
| Low physical activity |  | 2637 (67.08) |  | 2162 (66.32) |  | 475 (70.79) |  | 0.02 |
| DM family history |  | 363 (9.89) |  | 299 (9.78) |  | 64 (10.42) |  | 0.88 |
| Hypertension |  | 291 (25.64) |  | 403 (12.36) |  | 174 (25.93) |  | <0.001 |
| Dyslipidemia |  | 1679 (42.71) |  | 1295 (39.72) |  | 384 (57.23) |  | <0.001 |
| SBP (mmHg) |  | 114.43±15.43 |  | 113.18±14.87 |  | 120.51±16.65 |  | <0.001 |
| DBP (mmHg) |  | 76.38±10.21 |  | 75.60±10.06 |  | 80.18±10.08 |  | <0.001 |
| FBS (mg/dL) |  | 88.46±8.73 |  | 99.02±9.19 |  | 99.02±9.19 |  | <0.001 |
| Triglyceride (mg/dL) |  | 157.51±97.40 |  | 150.54±89.56 |  | 191.38±123.47 |  | <0.001 |
| HDL-C (mg/dL) |  | 42.06±10.78 |  | 42.10±10.78 |  | 41.84±10.78 |  | 0.56 |
| Anti-hypertensive drug use |  | 116 (2.95) |  | 81 (2.49) |  | 35 (5.22) |  | <0.001 |
| Lipid-lowering drug use |  | 52 (1.32) |  | 16 (2.38) |  | 16 (2.38) |  | 0.008 |
| MetS (JIS) |  | 1123 (28.57) |  | 747 (22.91) |  | 376 (56.04) |  | <0.001 |
| MetS (IDF) |  | 1000 (25.44) |  | 684 (20.98) |  | 316 (47.09) |  | <0.001 |
| cMetS-S |  | 0.0±1.0 |  | -0.1±0.98 |  | 0.49±0.93 |  | <0.001 |

The categorical and continuous variables were reported as count (percentage) and mean ± SD, respectively.
Abbreviations: cMetS-S, continuous metabolic syndrome severity score; DM, diabetes; SBP, systolic blood pressure; DBP, diastolic blood pressure; FBS, fasting blood sugar, HDL-C, high density lipoprotein cholesterol; MetS, metabolic syndrome; JIS, joint interim statement; IDF, International Diabetes Federation.
